# Benefits of Exome Sequencing in Children with Suspected Isolated Hearing Loss

**DOI:** 10.3390/genes12081277

**Published:** 2021-08-20

**Authors:** Roxane Van Heurck, Maria Teresa Carminho-Rodrigues, Emmanuelle Ranza, Caterina Stafuzza, Lina Quteineh, Corinne Gehrig, Eva Hammar, Michel Guipponi, Marc Abramowicz, Pascal Senn, Nils Guinand, Helene Cao-Van, Ariane Paoloni-Giacobino

**Affiliations:** 1 Division of Genetic Medicine, Geneva University Hospitals, 1205 Geneva, Switzerland; roxane.vanheurck@hcuge.ch (R.V.H.); mtcarminho@gmail.com (M.T.C.-R.); Emmanuelle.Ranza@medigenome.ch (E.R.); Lina.Quteineh@hcuge.ch (L.Q.); Corinne.Gehrig@hcuge.ch (C.G.); Eva.Hammar@hcuge.ch (E.H.); Michel.Guipponi@hcuge.ch (M.G.); Marc.Abramowicz@hcuge.ch (M.A.); 2 Ear-Nose-Throat/Head and Neck Surgery Division, Geneva University Hospitals, 1205 Geneva, Switzerland; Caterina.Stafuzza@hcuge.ch (C.S.); Pascal.Senn@hcuge.ch (P.S.); Nils.Guinand@hcuge.ch (N.G.); Helene.CaoVan@hcuge.ch (H.C.-V.)

**Keywords:** isolated hearing loss, deafness, genetics, molecular diagnosis, children, whole-exome sequencing

## Abstract

Purpose: Hearing loss is characterized by an extensive genetic heterogeneity and remains a common disorder in children. Molecular diagnosis is of particular benefit in children, and permits the early identification of clinically-unrecognized hearing loss syndromes, which permits effective clinical management and follow-up, including genetic counselling. Methods: We performed whole-exome sequencing with the analysis of a panel of 189 genes associated with hearing loss in a prospective cohort of 61 children and 9 adults presenting mainly with isolated hearing loss. Results: The overall diagnostic rate using exome sequencing was 47.2% (52.5% in children; 22% in adults). In children with confirmed molecular results, 17/32 (53.2%) showed autosomal recessive inheritance patterns, 14/32 (43.75%) showed an autosomal dominant condition, and one case had X-linked hearing loss. In adults, the two patients showed an autosomal dominant inheritance pattern. Among the 32 children, 17 (53.1%) had nonsyndromic hearing loss and 15 (46.7%) had syndromic hearing loss. One adult was diagnosed with syndromic hearing loss and one with nonsyndromic hearing loss. The most common causative genes were STRC (5 cases), GJB2 (3 cases), COL11A1 (3 cases), and ACTG1 (3 cases). Conclusions: Exome sequencing has a high diagnostic yield in children with hearing loss and can reveal a syndromic hearing loss form before other organs/systems become involved, allowing the surveillance of unrecognized present and/or future complications associated with these syndromes.

## 1. Introduction

Almost one in 500 infants is affected by hearing loss (HL) [1,2]. The prevalence increases dramatically with age in adults, and it has been estimated that approximately one-half of all adults aged between 60 and 69 years and 80% of those over 80 years old suffer from HL [3]. More than one-half of congenital or early-onset, bilateral sensorineural (SN) cases are believed to have a genetic cause, with the remainder either acquired or idiopathic [1,4]. Genetic etiologies are further divided into isolated or syndromic HL (associated with dysmorphic features and/or additional medical problems). However, it is often difficult to distinguish between syndromic and non-syndromic forms at an early age, as other signs and symptoms may appear only later in life [5]. Hearing screenings are recommended for newborns, as early detection and diagnosis of HL has been proven to improve health outcomes [6]. Universal newborn hearing screening was recommended in 1998 in the European Consensus Statement on Neonatal Hearing Screening in Newborns [7] and introduced in Switzerland in 1999 under the auspices of the “Swiss Working Group: Hearing Screening in Newborns”.

Screening in Switzerland is performed by estimating otoacoustic emissions (OAE) during the newborn’s stay at the maternity unit. If the OAE test fails in one or both ears, further evaluation is recommended, usually starting by repeating OEA measures the same or the following day. If OAEs are still undetectable uni- or bi- laterally after repeated measures, the infant is referred for additional investigations within the first months of life. Exclusion of congenital cytomegalovirus infection by polymerase chain reaction analysis on a “Guthrie” (‘blood spot’) card is part of the routine diagnostic evaluation. Ear-nose-throat (ENT) specialists determine the type and degree of HL through audiometry. In the event of rapidly progressive or sudden or unilateral deafness, ear imaging is performed using a computed tomography (CT) scan of the temporal bone in thin sections and magnetic resonance imaging (MRI) of the inner ear and auditory pathways [8].

Before the emergence and accessibility of broad genetic testing to identify syndromic HL forms, etiological assessment of HL often included additional examinations, such as thyroid assessment, electrocardiogram, urinary tract ultrasound and ophthalmologic examination. These examinations are no longer requested at the time of initial HL diagnosis, but they are performed if necessary, depending on the genetic assessment. In the absence of an environmental cause, international recommendations emphasize the importance of an early genetic analysis as a first-line assessment of a hearing disorder [5,8,9,10], as a molecular diagnosis can help guide management and counseling. However, one of the challenges of molecular analysis resides in the high degree of genetic heterogeneity [1]. Indeed, over 70 genes have been associated with isolated SN HL and with limited phenotypic clues. Thus, it is impossible to target a particular gene based on the phenotype alone. This may also hold true for syndromic cases as infants. Indeed at early age, HL may be the first sign of an underlying syndromic form, and therefore, infants affected by syndromic HL may be referred to a genetic consultation with isolated HL. Children affected by HL can greatly benefit from next-generation-sequencing in order to identify a genetic cause. To date, 30 genes are known to be associated with late onset or progressive HL [11]. As there is a high prevalence of HL in adults, it is important to identify those with a genetic etiology, as this might influence their management and permit early counseling. The purpose of this study was to describe the outcome of molecular analyses performed in 70 cases with a HL diagnosis, including a brief clinical description of confirmed cases.

## 2. Materials and Methods

### 2.1. Patients

A total of 61 children and 9 adults with an HL diagnosis were referred to the molecular laboratory of the Division of Medical Genetics at Geneva University Hospitals (Geneva, Switzerland) between January 2017 and December 2020. Following written informed consent obtained from all adult patients and the parents or guardians of children, as well as health insurance approval, patients underwent whole-exome sequencing (WES) with a bioinformatic analysis of HL and an ear malformation updated panel ranging from 172 to 189 genes, including *GJB2/GJB6.* Genes were selected according to the PanelApp classification of genes involved in hearing loss (Genomics England PanelApp. Available online: https://panelapp.agha.umccr.org/panels/209/ (accessed on 1 August 2021)). Of the 222 indicated genes, all green and litterature-relevant orange genes were chosen to build our in house gene panel (Table S1).

### 2.2. DNA

DNA from affected individuals and family members was extracted from whole blood. Exome sequencing was captured using one of the following kits according to the manufacturers’ recommendations: SureSelect QXT All Human Exon v5, v7 (Agilent Technologies, Santa Clara, CA, USA) or Twist Core exome + Refseq kits (Twist Biosciences, South San Francisco, CA, USA). Paired-end sequencing was carried out on a NextSeq500 Instrument (Illumina, San Diego, CA, USA). Targeted bioinformatic analysis of a panel of genes involved in HL was performed through locally-developed pipelines. Reads mapping and variant calling were performed using BWA V0.7.13, Picard V2.9.0 and GATK Haplotype-Caller V3.7 and annotated with annovar V2017-07-12 and UCSC RefSeq (V2018-08-10). The variants were searched for in various databases including dbSNP151, gnomAD 2.1, ClinVar 2018 and HGMD 2016. Pathogenicity prediction scores were assessed using dbscSNV and SpliceAI. Variant filtering and classification was performed based on the guidelines for the interpretation of sequence variants from the American College of Medical Genetics and Genomics (ACMG) and the Association for Molecular Pathology [12]. Sanger sequencing of custom-designed amplicons was used to confirm potentially disease-causing variants in probands and to perform segregation analysis. Pathogenicity scores were obtained for missense and splicing variants using SIFT, PolyPhen, MutationTaster, CADD, pAI, dbscSNV, spliceAI. Intermediate results were discussed in a multidisciplinary team including geneticists, biologists and ENT specialists. Copy number variation (CNV) detection on exome data was performed using an in-house read depth-based algorithm combining CoNIFER [13] and XHMM [14]. CoNIFER calculates normalized read-depth z-scores for each exon, generating a matrix of dimension *n* × M, with *n* being the number of captured exons and M the total number of samples. This matrix is then fed to XHMM, which uses hidden markov models to find streches of duplicated or deleted exons. Duplications and deletions overlapping genes of the hearing-loss panel are visually reviewed using a private instance of jBrowse [15], together with the per-exon read-depth z-scores. CNV calls detected by our algorithm were independently confirmed by multiplex ligation-dependent probe amplification (MLPA) analysis.

In Switzerland, patient access to WES is dependent on approval by the health insurance company. In some cases, *GJB2-GJB6* analysis is asked for beforehand, even if present in our exome gene panel, as it is one of the most frequently involved genes in non-syndromic HL [16]. Four additional children and one adult were found to carry *GJB2* mutations by this strategy and were therefore not included in our exome cohort.

## 3. Results

### 3.1. Cohort Descriptions

#### 3.1.1. Children

Sixty-one children (female, 26; male, 35; age range, 13 months to 18 years) and nine adults (female, 6; male, 3; age range, 34–78 years) benefited from a molecular analysis for HL. Most presented with SN HL (52 children (85.2%); 8 adults (88.9%)). Six children (9.8%) had mixed (conductive and SN) HL, two children (3.3%) and one adult (11.1%) had transmission HL, and one child had right SN HL and left mixed HL. HL severity among children was as follows: eight had mild HL (13.1%); 35 had moderate HL (57.4%); 10 had severe HL (16.4%); eight had profound HL (13.1%); and 6 showed progressive HL (9.8%). (Severity was defined as mild: 26–40 db hearing loss; moderate: 41–70 db hearing loss; severe: 71–90 db hearing loss and profound >91 db hearing loss).

The majority of children (53/61 (86.8%)) had bilateral HL. Congenital HL was diagnosed in 36/61 (59%) cases. Eleven (18%) children were diagnosed with prelingual HL (defined as identified at ≤1 year of age) and the remainder (14 patients (22.9%)) had postlingual HL (defined as identified at >1 year of age). Forty-one had no family history (67.2%) and only four probands (6.5%) were born from consanguineous parents. Of the 41 patients who underwent a CT scan and/or MRI investigation, 15 (36.6%) had middle/inner ear malformations. Twenty-one patients (34.4%) had other signs and symptoms in addition to HL (Table S2).

#### 3.1.2. Adults

Four adults had moderate HL (44.4%), four had severe HL (44.4%), and one had profound HL (11.1%); all cases showed progressive HL (Table S3). Three patients experienced a violent worsening of HL associated with upper airway infection for one patient and vertigo for the second case. The third patient did not recall any infection or vertigo associated with the onset of worsening of HL. All had bilateral HL at the time of consultation, but three patients had marked asymmetry at diagnosis. All were diagnosed with postlingual HL, but the age of onset was highly variable (8 to 65 years). Five patients had a family history (55.6%), three patients had no family history (33.3%), and one patient was adopted (no family history available). Two probands (22.2%) were born from consanguineous parents. Seven (77.8%) were investigated through CT scan and/or MRI, which revealed that four (44.4%) had middle/inner ear malformations. Interestingly, six of nine (66.7%) patients had additional symptoms to HL, with vertigo being the most frequent (Table S3).

#### 3.1.3. Patients Identified through Direct Sequencing of GJB2/GJB6

All four children that were diagnosed through direct sequencing of *GJB2/6* presented with SN, bilateral HL. Three had congenital HL and one was diagnosed with pre lingual HL. Three had severe HL and one had moderate HL. None were known with any family history or consanguinity. One adult was diagnosed through direct sequencing of *GJB2/6.* He displayed congenital, SN, bilateral profound HL. Three of the patient were males and two were females, out of wich one was an adult.

### 3.2. Molecular Results

#### 3.2.1. Children

Among the 61 cases investigated through WES, molecular confirmation was performed in 32 probands (52.5%) with the involvement of 22 different genes (Table S4). Five patients (patients 1, 33, 42, 46,51), showed a variant of unknown significance (VUS) (Table S5). Of note, patient 1 had a likely pathogenic de novo mutation in *COL4A5* and two missense variants in compound heterozygosity in *COL11A2* classified as VUS. (Tables S4 and S5). These variants did not fulfill ACMG criteria and were not counted or reported as positive, even if highly concordant with the patient’s phenotype. This was mostly due to the inherited status of the variant from a parent with normal audition or because segregation analysis was not possible.

Seventeen of 32 (53.1%) patients had autosomal recessive inheritance patterns; 14 (42%) had an autosomal dominant disorder, and one case had X-linked HL (Table S4). Among the 14 autosomal dominant cases, nine were reported de novo, three were inherited from a healthy parent, and one was inherited from an affected parent. One patient (# 21) had one HL variant (*POU4F3*) inherited from an affected father and a de novo incidental finding in *OPA1*. Another patient (# 24) had a de novo causative variant in *COL11A1* and an inherited *SMAD3* variant from an affected mother (Table S4).

**Figure 1:**
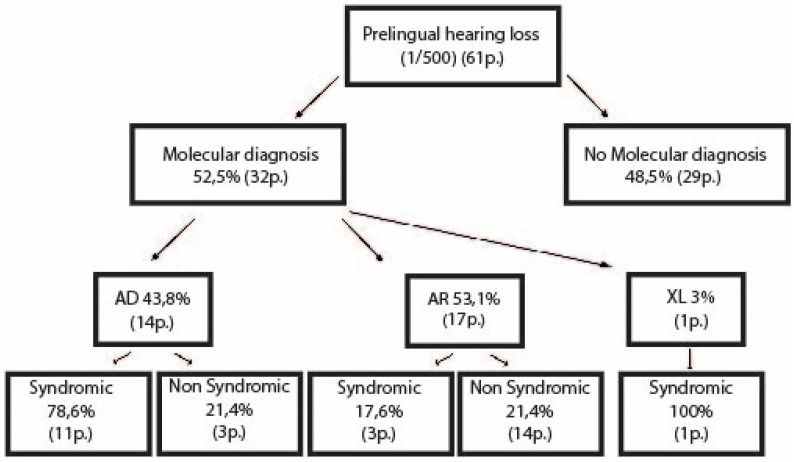
Description of the molecularly investigated child cohort. AD = autosomal dominant; AR = autosomal recessive; p. = patients; XL = X-linked.

**Figure 2:**
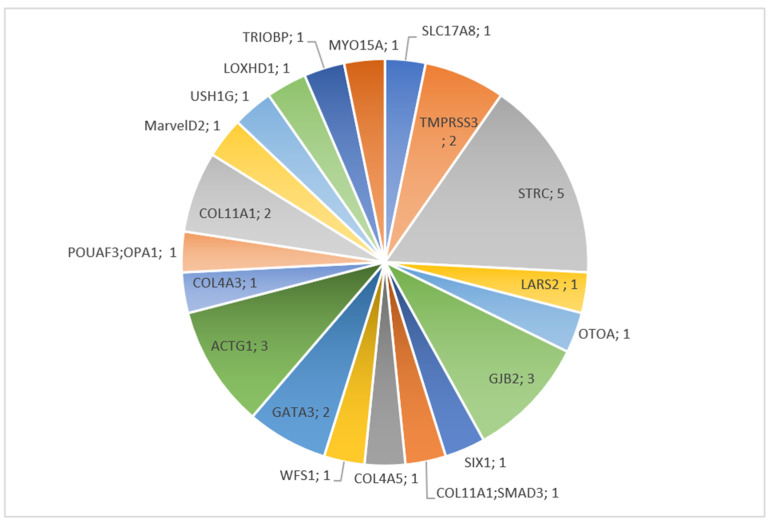
Distribution of genes identified through whole exome sequencing. Numbers represent the number of patients with the same molecular diagnosis.

Of the 32 children with a positive molecular diagnostic test, 17 (53.2%) had mutations in non-syndromic HL-associated genes, of which 14 were autosomal recessive (43.8%). Fifteen (46.9%) cases had pathogenic variants in syndromic HL-associated genes, of which 11 were transmitted in an autosomal dominant pattern (32.4%). Seven patients were counted as syndromic, but did not display any other sign apart from HL at the time of diagnosis (Figure 1). The most common HL causative genes were *STRC* (5 cases), *ACTG*1 (3 cases), *COL11A1* (3 cases), and *GJB2* (3 cases) (Table S4; Figure 2). Among these, only *COL11*A1 is responsible for both syndromic and non-syndromic HL. Four additional children were diagnosed with a *GJB2* mutation through direct sequencing (Table S6). Three patients were compound heterozygotes for a *STRC* point mutation and carried a *STRC* deletion on the other allele. Two other patients showed bi-allelic deletion of the *STRC* gene. One case was caused by a heterozygous gene conversion on one allele and CNV on the other; one patient showed heterozygous deletion of *COL11A1*. A total of seven cases were caused by CNV (21.9%).

#### 3.2.2. Adults

Among the nine adults that underwent molecular investigations, two had a molecular diagnosis (Table S4). One was diagnosed with neurofibromatosis type 2 and the other displayed a variant in the *COCH* gene. Both patients followed an autosomal dominant inheritance pattern. No family segregation was available and therefore it was not possible to conclude on a de novo or inherited status of these variants (Table S4). One patient was diagnosed with *GJB2* variants by direct sequencing (Table S6 (# 75)) and another showed a rare missense variant in the *TBC1D24* gene (Table S5 (# 64)). The latter variant was classified as a VUS based on ACMG criteria.

### 3.3. Brief Description of Individual Cases Confirmed by Molecular Diagnosis

#### 3.3.1. *COL4A5*

Patient 1. A six-year-old female presented with language delay, mild left and moderate right HL, associated with cochlear malformation. She had a conventional binaural behind-the-ear (BTE) hearing aid. No relevant family history was reported. WES identified a heterozygous likely pathogenic de novo missense variant in *COL4A5* (c.1525G > C, p.(Gly509Arg)). The *COL4A5* gene is associated with X-linked Alport syndrome characterized by SN HL, as well as ocular and kidney involvement. Females with *COL4A5* mutation can display HL, but it is usually less frequent and tends to occur in later life [17]. Nevertheless, a nephrology follow-up was organized, given the risk of renal complications in these patients [18,19]. WES also identified two missense variants in *COL11A2* classified as VUS and described below.

#### 3.3.2. *USH1G*

Patient 2. A 21-month-old female was diagnosed with profound bilateral SN HL with no relevant family history. WES identified a homozygous missense variant c.1373A > T, p.(Asp458Val) in *USH1G* and parental segregation was confirmed. *USH1G* is responsible for Usher syndrome type 1, an autosomal recessive condition that associates a congenital, profound SN HL, vestibular areflexia, and adolescent-onset retinitis pigmentosa. She benefited from a sequential bilateral cochlear implantation and a routine ophthalmologic evaluation [20]. The ophthalmological check-up revealed a pathological electroretinogram and close follow-up is ongoing.

#### 3.3.3. *GJB2*

Patient 3. A four-year-old male was diagnosed with bilateral, moderate, congenital SN HL, with no relevant family history. He had a conventional binaural BTE hearing aid. WES revealed a deletion c.35delG and a heterozygous missense c.101T > C, p.(Met34Thr) variant of *GJB2*. Parental segregation confirmed that mutations were in trans.

Patient 8. An eight-year-old male with congenital moderate SN HL, a conventional binaural BTE hearing aid and no relevant family history. An inner ear CT scan was normal. WES identified a compound heterozygous c.35del, p.(Gly12Valfs*2); c.139G > T, p.(Glu47*) in *GJB2*. Parental segregation confirmed that mutations were in trans.

Patient 20. A two-year-old boy with congenital severe, bilateral SN HL. He also presented palmoplantar keratoderma. Family history was unremarkable. An inner ear CT scan showed dilatation of the internal auditory canals and inner ear malformation. He benefited from a sequential bilateral cochlear implantation. WES revealed a pathogenic heterozygous de novo c. 223C > T, p.(Arg75Trp) in *GJB2*. Missense in this residue is associated with autosomal dominant HL and palmoplantar keratoderma [21,22].

#### 3.3.4. *SIX1*

Patient 4. An 11-year-old male with moderate SN HL on the right side and mixed, profound HL on the left side, and no family history of HL. He had bilateral inner ear malformations revealed by CT scan and left side congenital cholesteatoma. At physical examination, he presented with a pre-auricular pit on the left side. He had a conventional binaural BTE hearing aid. WES revealed a heterozygous de novo *SIX1* pathogenic missense mutation (c.386A > C, p.(Tyr129Ser)). *SIX1* is associated with branchiootorenal syndrome, which is characterized by branchial arch anomalies, hearing impairment (malformations of the auricle with pre-auricular pits and conductive or SN hearing impairment), and renal malformations [23]. Follow-up was completed with renal ultrasonography, which was normal.

#### 3.3.5. *LARS2*

Patient 5. An eight-year-old female with postlingual profound, bilateral, SN HL and no relevant family history. She benefited from a cochlear implantation on the right side. WES showed two compound heterozygous mutations (c.457A > C, p.(Asn153His) and c.1565C > A, p.(Thr522Asn)) in *LARS2.* Mutations were classified as likely pathogenic and pathogenic respectively. *LARS2* is associated with Perrault syndrome, which is characterized by SN HL in males and females and ovarian dysfunction in females. Pubertal development will be monitored in the future in order to induce puberty and permit normal bone mineralization. In the case of ovarian insufficiency, oocyte cryopreservation will be considered [24]. Follow-up was completed with ovarian ultrasonography and an endocrinological follow-up was organized.

#### 3.3.6. *ILDR1*

Patient 6. A 10-year-old male with congenital, profound, bilateral SN HL. He benefited from a unilateral cochlear implantation. Apart from being born into a consanguineous union, he had no other relevant family history. WES identified a homozygous nonsense mutation (c.942C > A, p.(Cys314*)) in *ILDR1*, classified as pathogenic. Mutations in this gene are known to cause a prelingual, nonprogressive, nonsyndromic form of SN deafness [25].

#### 3.3.7. *ACTG1*

Patient 7. A five-year-old male with postlingual unilateral (left) mild mixed HL. He had no relevant family history and no other health problems. The CT scan showed an uncus malformation on both sides, but normal inner ears. WES identified a heterozygous, likely pathogenic de novo mutation, c.440G > A, p.(Arg147His) in the *ACTG1* gene.

Patient 16. A 15-year-old female with postlingual, bilateral, moderate SN HL. She had a binaural conventional BTE hearing aid. No relevant family history was noted. WES revealed a heterozygous, likely pathogenic de novo mutation, c.826G > A, p.(Glu276Lys) in *ACTG1*.

Patient 30. A 17-year-old male with an initial mild SN postlingual HL that had progressed to moderate HL. Both his paternal grandmother and his maternal grandfather showed late onset HL. He had a bilateral conventional BTE hearing aid since the age of 16 years. WES identified a heterozygous, pathogenic de novo mutation, c.830C > T,p.(Thr277Ile) in *ACT*G1.

*ACTG1* variants are responsible for DFNA20/DFNA26, usually associated with postlingual and progressive SN HL and a type 2 Baraitser-Winter syndrome. Our patients did not have any syndromic features to date and thus we considered that these mutations were related to autosomal dominant deafness 20/26 (MIM: 604717) [26,27,28].

#### 3.3.8. *GATA3*

Patient 9. A five-year-old male with congenital moderate SN HL and bilateral renal cysts. He had a binaural conventional BTE hearing aid. No relevant family history was noted. WES identified a pathogenic heterozygous de novo mutation c.778 + 1G > A, p.?, in *GATA3*.

Patient 19. An 18-month-old male with a similar history to patient 9. He presented bilateral moderate SN HL, unilateral kidney dysplasia and cystic dilatation of the rete testis of the right testis. He had a bilateral conventional BTE hearing aid. WES identified a pathogenic, de novo heterozygous c.431delG, p.(Gly144Alafs*51) in *GATA3*.

*GATA3* is associated with HDR syndrome, i.e., hypoparathyroidism, SN deafness and renal dysplasia. Hypoparathyroidism can appear later in life and both patients are under endocrinological surveillance [29].

#### 3.3.9. *SLC17A8*

Patient 10. A four-year-old-male with congenital bilateral, moderate SN HL. His maternal grandmother was also known for HL, without further information. He had a bilateral conventional BTE hearing aid. WES identified a likely pathogenic, heterozygous mutation (c.634C > A, p.(Pro212Thr)) in *SLC17A8* inherited from his mother with normal audition.

*SLC17A8* is known to be associated with highly variable non-syndromic HL. Affected male members are reported with earlier onset and a more severe phenotype [30].

#### 3.3.10. *LOXHD1*

Patient 11. An eight-year-old female with bilateral moderate SN HL and no relevant family history. She had a bilateral conventional BTE hearing aid. WES identified a pathogenic homozygous mutation (c.3061 + 1G > A, p.?) in *LOXHD1*. Parental segregation was confirmed in the mother, but was not available for the father. *LOXHD1* is associated with autosomal recessive bilateral SN HL, which can be progressive. Mutations in this gene have also been recently associated with late-onset Fuchs corneal dystrophy and therefore ophthalmological surveillance was recommended [31].

#### 3.3.11. *OTOA*

Patient 17. A three-year-old female with bilateral mild-to-moderate congenital SN HL born to consanguineous parents without any relevant family history. WES identified a paternal gene conversion between *OTOA* gene and *OTOAP1* pseudogene and a maternal deletion of *OTOA*. Gene conversion between *OTOA* and its pseudogene *OTOAP*1 is a known mechanism leading to the generation of a pathogenic *OTOA* allele [32]. Exons 20 to 28 of the *OTOA* gene are located in a 68 kb region that was segmentally duplicated during evolution and resulted in the emergence of the *OTOAP1* pseudogene located 820 kb upstream of OTOA. Therefore, these genes share a high level of homology (>99%). In our patient, gene conversion occurred between the exon 20–21 of the *OTOA* gene replaced by exon 1 and 2 of the pseudogene *OTPAP1* [33]. This gene conversion is expected to result in a premature stop codon that would either result in a truncated protein or an absence of protein through mRNA nonsense-mediated decay. These findings were confirmed by polymerase chain reaction/Sanger sequencing and MLPA. Family segregation confirmed the inheritance of each allele from one of the parents. *OTOA* is related to autosomal recessive non-syndromic HL [34].

#### 3.3.12. *WSF1*

Patient 18. A two-year-old female who suffered from congenital bilateral moderate SN HL. HL was progressive and she had profound bilateral deafness. No relevant family history was noted. She benefited from sequential bilateral cochlear implantation. WES identified a pathogenic de novo heterozygous mutation (c.2051C > T, p.(Ala684Val)) in *WSF1*.

*WSF1* is associated with an autosomal dominant Wolfram-like syndrome, which associates progressive HL, optic atrophy and, later, diabetes mellitus. After diagnosis, the patient had an ophthalmological evaluation that revealed partial bilateral optic atrophy. Endocrinological follow-up was organized [35].

#### 3.3.13. *STRC*

Patient 12. A seven-year-old male was diagnosed with mild bilateral SN HL when he started elementary school. He had a conventional binaural BTE hearing aid. MRI was normal, and there was no relevant family history. WES identified a compound heterozygous *CKMT1B, STRC, CATSPER2* deletion, confirmed by MLPA (chr15:g.(43851199_43890333)_(43940820_44038794)del) and (c.4917_4918delACinsCT, p.(Leu1640Phe)) in *STRC*. Family segregation was confirmed.

Patient 13. A seven-year-old male with postlingual, moderate bilateral SN HL. The audiogram showed a “U-curve”. He had a conventional binaural BTE hearing aid. MRI was normal with no relevant family history. WES identified a compound heterozygous *CKMT1B, STRC* deletion and a *CKMT1B*, *STRC*, *CATSPER2* deletion). This result was confirmed by MLPA (chr15:g.[(43851199_43890333)_(43897676_43924279)del];[(43851199_43890333)_(43940820_44038794)del] and family segregation.

Patient 14. A nine-year-old female with moderate prelingual SN bilateral HL and no relevant family history. WES identified a compound heterozygous c.4425G > C, p.(Trp1475Cys) in *STRC* and *CKMT1B, STRC, CATSPER2* deletion confirmed by MLPA (chr15:g.(43851199_43890333)_(43940820_44038794)del) and family segregation confirmed bi-allelic inheritance.

Patient 15. A nine-year-old male with congenital moderate bilateral SN HL and no relevant family history. He had a conventional binaural BTE hearing aid. WES identified a homozygous deletion of *CKMT1B, STRC, CATSPER2* confirmed by MLPA (chr15:g.(43851199_43890333)_(43940820_44038794)del) and family segregation confirmed bi-allelic inheritance.

Patient 22. A 14-year-old female with moderate, prelingual bilateral SN HL. Her father suffered from moderate bilateral HL and her uncle suffered from unilateral moderate HL. She had a conventional BTE hearing aid since the age of one year. WES identified a heterozygous deletion of *CKMT1B and STRC* and probably *CATSPER2* confirmed by MLPA (chr15:g.(43′851′199_43′890′391)_(?_44′038′820)del), as well as a heterozygous (c.4837G > T,p.(Glu1613*)) mutation in the *STRC* gene. Family segregation confirmed the inheritance of each allele from a healthy parent.

*STRC* alterations cause autosomal recessive nonsyndromic SN deafness type-16. HL starts usually during childhood (birth to the age of 10 years). Contiguous gene deletion syndrome on chromosome 15q15.3, including *STRC* and *CATSPER2*, as identified in patient 15, is responsible for a deafness-infertility syndrome. This syndrome is characterized by early-onset deafness in both males and females and associated with infertility in males [36].

#### 3.3.14. *POU4F3* and *OPA1*

Patient 21. A six-year-old female with a moderate bilateral SN HL detected at elementary school screening. Her audiogram was “spoon”-shaped. She had a conventional binaural BTE hearing aid. Family history was positive on her father’s side (paternal uncle, grandfather and great-grandfather affected with adult onset moderate HL). Her father had encountered difficulties discriminating sounds since childhood, but he reported his audiological evaluation as normal at 18 years of age. WES revealed a heterozygous pathogenic mutation (c.502del, p.(Ala168Profs*36)) in *POU4F3* inherited from the father and a likely pathogenic, heterozygous de novo mutation (c.1118C > G, p.(Ser373Cys)) in *OPA1*, never reported previously.

*POU4F3* is associated with autosomal dominant deafness type 15, a progressive form of nonsyndromic SN HL. Onset is postlingual, usually between the second and sixth decades of life. Intrafamilial variability has been reported [37]. *OPA1* is related with optic atrophy and optic atrophy plus syndrome [38]. Multisystem neurological disease involving optic atrophy, deafness and neuromuscular complications is associated with all types of mutations. However, optic atrophy plus syndrome is more frequent with a missense mutation in *OPA1* gene, while classic optic atrophy is mostly associated with deletion. Both groups of mutations are most frequently observed in the GTPase domain of the *OPA1* gene [39]. The mutation carried by our patient was located in this GTPpase domain. Although she had a normal ophtalmological and neurological evaluation, close follow-up was recommended as the signs and symptoms can be progressive and highly variable, with a mean onset around 10 years of age [39,40].

#### 3.3.15. *COL11A1*

Patient 23. A three-year-old male was referred to an ENT specialist due to delayed speech. Audiograms showed mild prelingual SN bilateral HL. He benefited from neuropediatric evaluation because of interaction difficulties, excessive shyness and motor coordination problems. The family history revealed that his two brothers, his mother, two of his maternal aunts and his grandmother suffer from HL. No one was wearing hearing aids. Age of onset was highly variable (35 years for his mother and 10–15 years for his brothers). HL appeared to be isolated in the family and, in particular, there was no history of cleft palate. WES revealed a large heterozygous pathogenic deletion of *COL11A1* (chr1:g.[(103388956_103400026)_(104094395_?)del]) inherited from his mother and present in both of the patient‘s brothers. The patient and his brothers underwent ophtalmological investigations, which were completely normal.

Patient 26. A 15-year-old male with congenital bilateral moderate SN HL. His grandmother had a very late onset history of HL. Hearing aids were added sequentially (right ear at 2 years and left ear at 4 years). WES revealed a heterozygous likely pathogenic deletion of a splicing site in *COL11A1* (c.4519-2Adel,p.?) inherited from his apparently asymptomatic mother who never benefited from an audiological examination. Close ophtalmological follow-up was recommended, even if HL seemed isolated.

#### 3.3.16. *COL11A1* and *SMAD3*

Patient 24. An 18-year-old female with congenital bilateral moderate SN HL who had been wearing hearing aids since the age of 4 years. She was born with a cleft palate and was highly myopic. She reported bruising easily and chronic knee pain related to recurrent patella luxation. The family history revealed that her mother needed surgical correction of cervical vertebrae, complicated by severe hemorrhage, but without further information. She had two healthy brothers. All of her mother’s pregnancies were uncomplicated. WES revealed a heterozygous pathogenic de novo variant in *COL11A1* (c.4547G > T, p.(Gly1516Val)) and a likely pathogenic heterozygous variant in *SMAD3* (c.3G > A (p.Met1?)) inherited from her mother.

*COl11A1* is associated with Marshall syndrome and Stickler syndrome type II or autosomal dominant deafness type 37. Marshal syndrome is characterized by dysmorphic signs (microcretrognathia, long philtrum, robin sequence) and key clinical features, such as cleft palate, myopia and SN HL. Main complications are vitreoretinal degeneration, glaucoma and retinal detachment. Stickler syndrome is characterized by dysmorphic signs (micrognathia and Pierre Robin sequence with cleft palate), SN HL, early onset myopia, glaucoma and a risk of retinal detachment. Joint hypermobility is common and associated with an increased risk of early onset arthrosis [41].

Patients 23 and 26 were affected only by HL, while patient 24 displayed clear signs of Stickler syndrome. It has recently been highlighted that *COL11A1* is associated with nonsyndromic HL and should be included in nonsyndromic HL gene panels [42].

*SMAD3* is associated with Loeys-Dietz syndrome type 3, characterized by cardiac malformation (mitral valve prolapse, aortic insufficiency, left ventricular hypertrophy) and an increased risk of aortic aneurysm and dissection, arterial aneurysm and arterial tortuosity, pectus deformity, and an increased risk of internal organ ruptures [43].

Patient 24 underwent extensive vascular investigations and had no signs of vascular involvement or characteristic Loeys-Dietz dysmorphic features. However, she did describe bruising easily, had fair skin and long fingers. Due to her young age, we proposed to introduce a regular vascular follow-up.

#### 3.3.17. *TRIOBP*

Patient 25. A five-year-old female was referred to the ENT department for profound, seemingly isolated and congenital HL. No relevant family history was noted, but her parents were consanguineous. WES revealed homozygous pathogenic duplication in the *TRIOBP* gene (c.3214dup, p.ArgArg1072Profs*12). Family segregation confirmed a bi-allelic inheritance.

The *TRIOBP* gene is associated with nonsyndromic autosomal recessive HL, which is usually bilateral and prelingual [44].

#### 3.3.18. *TMPRSS3*

Patient 27. A nine-year-old female was referred to the ENT department due to severe bilateral progressive HL. She had been wearing regular BTE hearing aids since the age of 8 years with poor results. The family history was unremarkable. WES revealed compound heterozygosity in the *TMPRSS3* gene (c.400 A > T, p.(Lys134*); c.646C > T, p.(Arg216Cys)); both variants were reported as pathogenic. Family segregation confirmed that each variant was inherited from a healthy parent.

Patient 29. A three-year-old male was referred to the ENT department due to speech delay. OAEs at birth were reported normal, but a hearing evaluation revealed severe bilateral “ski slope” pattern SN HL. He had a sister whose audition was in the normal range. His father reported unilateral HL since childhood. Hearing aids were implemented since the diagnosis. WES revealed compound heterozygosity in the *TMPRSS3* gene (c.916G > A, p.(Ala306Thr); (c.749delT,p.(Leu250Argfs*25)); both variants were reported as pathogenic. Family segregation confirmed the bi-allelic inheritance of the variants.

The *TMPRSS3* gene is associated with nonsyndromic recessive SN HL type 8/10. HL can be pre- or post- lingual, depending on the type of mutation, and HL has been described as isolated [45].

#### 3.3.19. *COL4A3*

Patient 28. A 10-year-old male with mild bilateral SN HL. The family history revealed that both his father and grandfather suffered from mild HL, but progressive for his father. He had one healthy brother. We could not find an audiological examination (OEA) performed at birth, but screening at the age of 5 years was pathological. WES revealed a heterozygous, likely pathogenic mutation in *COL4A3* inherited from his father (c.4826G > A, p.(Arg1609Gln)).

*COL4A3* mutations are associated with several phenotypes, such as Alport syndrome that associates renal failure, variable HL (which can be of late onset) and ocular involvement, including cataract and retinopathies. Heterozygous mutation in the *COL4A3* gene responsible for autosomal dominant Alport syndrome might also generate isolated HL or HL with ocular involvement in some carriers [46]. Neither our patient nor his father showed any sign of kidney or ocular involvement, but close follow-up was organized as phenotypic variability has been described, even in the same family [46].

#### 3.3.20. *MARVELD2*

Patient 31. A 16-year-old male presenting with severe bilateral HL. He wore regular BTE hearing aids. In addition, he suffered from cholestatic hepatopathy since infancy, as well as hyperactivity and impaired concentration. The family history was unremarkable. His parents of European descent were not related. WES revealed a homozygous pathogenic mutation in the *MARVELD2* gene (c.1331 + 2T > C, p.(?)).

*MARVELD2* is associated with autosomal recessive nonsyndromic HL type 49 more often found in the East Caucasian population. No association with hepatic involvement in this patient could be demonstrated. However, MARVELD2 is a tight junction protein and therefore we might reconsider this statement in the years to come [47]. Indeed we might stretch out that a defect in tight junction protein could easily affect the integrity of the epithelia, and therefore, the function of the organ.

#### 3.3.21. *MYO15A*

Patient 32. A two-year-old male with profound bilateral congenital HL. At birth, he presented with hypothyroidism and an atrial septal defect. He benefited from a sequential bilateral cochlear implant (Nov 2020, right side; Feb 2021, left side). He had one older sister without any hearing impairment. His paternal grandfather was reported with mild HL and his great-granduncle with very early HL (without further information). His parents have normal audition. WES revealed a homozygous variant in the *MYO15A* gene. Family segregation confirmed inheritance of each of the variants from a healthy parent.

*MYO15A* gene is associated with autosomic recessive severe nonsyndromic HL type 3. HL is described as congenital and severe to profound [48].

#### 3.3.22. *NF2*

Patient 62. A 40-year-old male presented with unilateral progressive HL and bilateral tinnitus. MRI revealed bilateral schwannoma, which raised the hypothesis of neurofibromatosis type 2. Exome sequencing revealed a heterozygous pathogenic variant in the *NF2* gene (c.1579G > T, p.(Glu527*)), which confirmed the diagnosis.

A *NF2*-related syndrome is characterized by the progressive appearance of vestibular schwannomas, which are usually bilateral and responsible for HL, and may or may not be associated with tinnitus. Schwannomas may also develop on other cranial, spinal or peripheric nerves with related symptoms. Some patients may develop intracranial or intraspinal meningiomas or a malignant tumor of the nervous system (ependymoma). Ophtalmological involvement, including cataract and loss of visual acuity, is quite common. Seventy percent of *NF2* patients have cutaneous tumors [49].

#### 3.3.23. *COCH*

Patient 63. A 66-year-old female with bilateral severe HL since 30 years. Lately, she noticed worsening of hearing impairment and bilateral vestibular areflexia. She wore regular hearing aids. Her sister had bilateral HL and wore hearing aids. Her brother and father displayed late-onset HL, but less severe. Her son is being evaluated for HL. WES revealed a heterozygous pathogenic mutation in the *COCH* gene (c.341T > C, p.Leu114Pro). The mutation associated with the *COCH* gene leads to progressive bilateral HL with autosomal dominant transmission. Vestibular involvement is frequent. Onset is described from 20 to 60 years of age [50].

### 3.4. Variant/s of Unknown Significance (VUS)

Patient 1 (described previously). In addition to the *COL4A5* variant, she was found to carry two missense variants in compound heterozygosity in *COL11A2* classified as VUS. Mutations in *COL11A2* are known to be responsible for autosomal dominant and autosomal recessive nonprogressive profound, congenital or prelingual HL. This gene is also associated with a syndromic HL, otospondylomegaepiphyseal dysplasia [51]. To date, our patient does not present any other signs and symptoms suggestive of a collagenopathy.

Patient 33. A 12-year-old male with prelingual bilateral severe SN HL. His younger brother was also affected. They had no other signs and symptoms. WES identified compound heterozygous mutations (c.641G > A, p.(Arg214His)) and (c.643T > G, p.(Trp215Gly)) classified as VUS in *TBC1D24*. Both variants were also present in the affected brother and family segregation was confirmed.

Patient 64. A 33-year-old female referred to the ENT department due to bilateral HL since the age of 18 years, with moderate, but progressive bilateral SN HL. The family history revealed that her father suffered from late-onset unilateral HL. She was born to a consanguineous union. WES revealed a heterogygous mutation in *TBC1D24* (c.418 C > G p.(Leu140Val)) classified as a VUS.

*TBC1D*24 has been described with autosomal recessive and autosomal dominant HL.

Autosomal recessive *TBC1D24*-related syndromes show a marked phenotypic pleiotropy with multisystem involvement. The severity spectrum ranges from isolated deafness to benign myoclonic epilepsy restricted to childhood with complete seizure control and normal intellect, to early-onset epileptic encephalopathy with severe developmental delay and early death. There is no distinct phenotypic correlation with the pathogenic variant type or location as yet, but patterns are emerging [52]. Autosomal dominant *TBC1D24*-related syndromes are marked by adult onset and progressive HL [53]. For both families of patients 33 and 64, the variants did not fulfill ACMG criteria and were not counted as positive results [12].

Patient 42. An 11-year old female with congenital bilateral non-syndromic SN HL. Neonatal hearing screening was abnormal and she underwent a cochlear implant. The family history revealed that she was born to a consanguineous union (2nd degree cousins), but with no history of HL. Her parents were of Egyptian origin. WES revealed a heterozygous variant in the *CDH23* gene. This latter gene is associated with autosomal recessive HL, autosomal dominant Usher syndrome 1D or autosomal recessive/digenic Usher syndrome [54,55]. A family segregation study was not possible and therefore pathogenicity through segregation could not be concluded. As ACMG criteria were not met, the variant was classified as a VUS [12], The patient will be re-evaluated on a regular basis.

Patient 46. A 14-year-old male with HL detected at the age of 3 years who wore regular BTE hearing aids since then. At age 12, he benefited from a cochlear implant. He was diagnosed with attention deficit hyperactivity disorder and benefits from special support at school. The family history was unremarkable. WES revealed two variants in *PCDH15* (c.4885delA, p.S1629fs & c.964T > A, p.Ser322Thr) and two variants in *USH2A* (c.13133C > T, p.Pro437Leu & c.6800C > T, p.Pro2267Leu). Family segregation confirmed the position in cis of all variants inherited from the healthy mother. All variants were classified as VUS.

*PCDH15* gene is associated with autosomal recessive HL type 23 and autosomal recessive/digenic Usher syndrome [55,56]. *USH2A* gene is associated with autosomal recessive Usher syndrome type 2A and retinitis pigmentosa 38 [55,57].

Patient 51. A 10-year-old female presenting with auditory neuropathy resulting in moderate HL. An attempt with hearing aids was not successful. The family history was unremarkable. WES revealed a heterozygous variant in the *OSBPL2* gene (c.852_854delTATinsATG, p.(Phe284_Met285delinsLeuTrp)) inherited from the healthy mother. Therefore, this variant did not fulfill ACMG criteria and was not considered as a positive result [12].

The *OSBPL2* gene is responsible for autosomal dominant HL with high variability in terms of age of onset (5–32 years) and expressivity; HL is usually progressive [58].

### 3.5. Molecular Results through Direct Sequencing of GJB2-GJB6

Patient 71. A three-year-old male with congenital severe bilateral SN HL and no relevant family history. Direct sequencing of *GJB2* and *GJB6* revealed a homozygous deletion in the *GJB2* gene (c.35delG, p.(Gly12Valfs*2).

Patient 72. An eight-year old male with prelingual severe bilateral SN HL and no relevant family history. Direct sequencing of *GJB2* and *GJB6* revealed a homozygous deletion in the *GJB2* gene (c.35delG, p.(Gly12Valfs*2). Family segregation was confirmed.

Patient 73. A five-year-old female with moderate congenital SN HL. Her mother had moderate-to-high frequency HL. She wore regular bilateral BTE hearing aids. Direct sequencing of the *GJB2* and *GJB6* locus revealed a compound heterozygous mutation in the *GJB2* gene (c.59T > C, p.Ile20Thr and c.109G > A,p.Val37Ile). Family segregation was confirmed.

Patient 74. A 6 year-old male with prelingual moderate bilateral SN HL and no relevant family history. Direct sequencing of *GJB2* and *GJB6* locus revealed a homozygous mutation in the *GJB2* gene (c.269T > C, p.(Leu90Pro). Family segregation was confirmed.

Patient 75. A 20-year old female with congenital severe HL. She wore regular BTE bilateral hearing aids and was referred to the genetics department because of her wish to conceive a child. She had two sisters with severe bilateral HL who also wore hearing aids. She had also one sister and one brother without any hearing impairment. Direct sequencing of the *GJB2* locus revealed a homozygous deletion in the *GJB2* gene (c.35delG, p.(Gly12Valfs*2).

## 4. Discussion

WES performed on a cohort of 61 children and 9 adult patients with HL identified a genetic etiology in 52.5% of children and 22.2% of adults. Our diagnostic yield in the child cohort was within the upper range (10–80%) of results published in the literature [1,2,59,60]. A high diagnostic yield from genomic testing has been associated with the inclusion of patients with early onset HL and suspected genetic syndromes, but who have not undergone any previous genetic testing for common genes, such as *GJB2-GJB6*. In our cohort, only three patients showed a *GJB2* mutation revealed by exome sequencing. When including the four children excluded from our exome cohort, our diagnostic rate reached 55.4% in this population. The inclusion of greater numbers of deafness genes and the addition of CNV analysis can also increase the diagnostic yield [60]. In our cases, we used large gene panels and pipeline analysis optimized to identify CNV. Seven cases were caused by CNVs detected by this algorithm, representing a substantial proportion of our cohort (21.9%). Therefore, it is important that the pipeline allows for the detection of such CNVs [14,61].

The genetic causes of hearing loss have also been explored in different adult populations [62,63,64]. Epidemiological studies focusing on the heritability of adult onset HL ranked it as between 19–53% in twin studies, without differentiation between monogenic and polygenic rates [65]. However, a recent study suggests that the diagnostic rate can be very high if patients are carefully selected [63,66]. In our cohort, the diagnostic rate was 22.2%. As we only included 9 adults with HL, this diagnostic rate has to be re-evaluated in a larger cohorts. A molecular diagnosis will help the medical team to adapt follow-up and provide appropriate genetic counseling.

Diagnostic rates of up to 60–80% are expected in patients with suspected autosomal recessive non-syndromic congenital deafness. In our cohort of molecularly diagnosed patients, 43.75% were autosomal recessive non-syndromic cases (14 patients); three patients with autosomal recessive inheritance were syndromic. In total, 53% of patients had autosomal recessive inheritance patterns and 43.75% had autosomal dominant transmission, of which 11 were syndromic. One patient showed an X-linked inheritance pattern, which corresponds to expected rates [59]. Both adults with a confirmed molecular diagnosis showed an autosomal dominant disorder. Autosomal dominant HL, especially later onset HL [9,10,67], is associated with genes that show incomplete penetrance and high variability [11] and this is therefore not surprising. Our rate of syndromic HL in children (46.9% syndromic) is slightly higher compared with those reported in the literature for a Caucasian population (30%) [10,11,16]. Among the 15 patients with syndromic HL, nine had other signs and symptoms at the time of diagnosis. For two cases, these additional signs were discovered thanks to the investigations launched after molecular diagnosis. The seven patients without any additional sign at the time of diagnosis are being closely followed-up according to molecular diagnosis recommendations. Molecular identification of these patients is extremely important as follow-up and treatment can be adapted in order to prevent potential complications.

Mutations in the *GJB2* gene are among the most frequent causes of non-syndromic congenital HL, with a variable range depending on ethnic variations (8% to 42.32%) [11,16]. The second most frequent non-syndromic HL-associated genes are *SLC26A4* and *OTOF*. In our cohort, we found *STRC* (five cases) to be the most frequent cause of non-syndromic congenital HL. Other genes that were most often altered in our population were *ACTG1* (*n* = 3), *COL11A1* (*n* = 2) and *GJB2* (*n* = 3). We did not identify any patient with *SLC26A4* and *OTOF* variants. Interestingly, our data expand the phenotype of already known HL genes, and we report two patients with non-syndromic hearing loss and a pathogenic de novo variant in *ACTG1*, responsible for DFNA20/DFNA26 and type 2 Baraitser-Winter syndrome. Therefore, our results contribute to expand the genotypic spectrum of *ACTG1*, which is associated with postlingual progressive SN HL [11,27,68].

We reported two patients with a *TMPRSS3* variant, which is a rare cause of non-syndromic HL in Caucasian patients (<1% vs. 6.3% in our cohort), but more frequent among Pakistani (1.8%), Tunisian (5%), Korean (5.9%), and especially Turkish patients (12%). Patient 27 is of Italian origin and patient 29 has French origins, thus suggesting that *TMPRSS3* mutations might be more frequently involved in non- syndromic HL than reported in the literature, even in Caucasian patients [11,45,69]. As shown previously, the local epidemiology and diagnostic rate vary widely regarding ethnicity [11,70,71,72]. Geneva is a city located at the crossroads of Europe and is known for its mixed ethnicity and thus our rates probably do not reflect a classical Western Europe Caucasian population epidemiology.

VUS were identified in five children (8%) and one adult (11.1%). We recommend the regular re-analysis of noninformative exomes and exomes containing VUS every 18 to 24 months after the first analysis. As WES data is stored in our bioinformatics department, re-analysis of these exomes with newly-described genes can be easily performed. Two cases have potentially two molecular diagnoses for HL. Of note, these findings can make genetic counseling more difficult and should be handled with care. Reported rates of dual diagnosis are around 1% to 4% [11,73]. In our cohort, two patients had a dual diagnosis (3.2%), which is concordant with the reported data in the published literature. No secondary accidental findings were identified as bioinformatic analyses were centered on the 189-gene panel for HL and ear malformations.

## 5. Conclusions

In conclusion, exome DNA sequencing and analysis of pathology-related gene panel(s) has become the gold standard for the investigation of HL. Our results emphasize the advantages of a global approach with careful variant and case discussion involving a multidisciplinary team to obtain a genetic diagnosis of SN HL. In addition to its undeniable value in clinical practice, with a 50–60% genetic diagnostic yield for SN HL (including *GJB2/GJB6* alterations), it improves prognostic accuracy, as well as genetic and reproductive counseling. Importantly, this approach can also reveal clinically-relevant undiagnosed syndromes, thus changing the outcome of the disorder and avoiding the occurrence of preventable complications.

## Data Availability

Reported genetic results can be found in ClinVar with accession number #SCV001739291 #SCV001745836 #SCV001745837 #SCV001739292 #SCV001745838 #SCV001745839 #SCV001739293 #SCV001739294 #SCV001739295 #SCV001739296 #SCV001739297 #SCV001739298 #SCV001745840 #SCV001745842 #SCV001745841 #SCV001739299 #SCV001739300 #SCV001745843 #SCV001739301 #SCV001739302 #SCV001739303 #SCV001739304 #SCV001739305 #SCV001745844 #SCV001745845 #SCV001739306 #SCV001739307 #SCV001739308 #SCV001739309 #SCV001745846 #SCV001745847 #SCV001745848 #SCV001739310 #SCV001745849 #SCV001745850 #SCV001745851 #SCV001739311 #SCV001739312 #SCV001745852 #SCV001745853 #SCV001745854 #SCV001745855 #SCV001745856 #SCV001739313 #SCV001739314 #SCV001739315 #SCV001739316 #SCV001745853 #SCV001745854 #SCV001745855 #SCV001745856 #SCV000999820.1 #SCV000999821.1 #SCV000999826.1 #SCV000897954.1 #SCV000897964.1 #SCV000897965.1. OTOA gene conversion is described in doi:10.1002/humu.24167.

## Supplementary Materials

The following are available online at www.mdpi.com/article/10.3390/genes12081277/s1, Table S1: In house hearing loss gene panel, Table S2: Phenotype children, Table S3: Phenotype Adults, Table S4: Molecular results, Table S5: Molecular results VUS, Table S6: GJB2 direct sequencing.

## Abbreviations

BTE	behind-the-ear
CNVs	copy number variant/s
CT	computed tomography
ENT	ear-nose-throat
HL	hearing loss
MLPA	multiplex ligation-dependent probe amplification
MRI	magnetic resonance imaging
OEA	otoacoustic emissions
SN	sensorineural
VUS	variant of unknown significance
WES	whole-exome sequencing

## References

[1] Morton C.C., Nance W.E. (2006). Newborn hearing screening—A silent revolution. N. Engl. J. Med..

[2] Morton N.E. (1991). Genetic epidemiology of hearing impairment. Ann. N. Y. Acad. Sci..

[3] Jilla A.M., Reed N.S., Oh E.S., Lin F.R. (2021). A Geriatrician’s Guide to Hearing Loss. J. Am. Geriatr. Soc..

[4] Marazita M.L., Ploughman L.M., Rawlings B., Remington E., Arnos K.S., Nance W.E. (1993). Genetic epidemiological studies of early-onset deafness in the U.S. school-age population. Am. J. Med. Genet..

[5] Smith R.J., Bale J.F., White K.R. (2005). Sensorineural hearing loss in children. Lancet.

[6] Nelson H.D., Bougatsos C., Nygren P., 2001 US Preventive Services Task Force (2008). Universal newborn hearing screening: Systematic review to update the 2001 US Preventive Services Task Force Recommendation. Pediatrics.

[7] Grandori F. (1999). The European Consensus Development Conference on Neonatal Hearing Screening (Milan, 15–16 May 1998). Arch. Otolaryngol. Head Neck Surg..

[8] Liming B.J., Carter J., Cheng A., Choo D., Curotta J., Carvalho D., Germiller J.A., Hone S., Kenna M.A., Loundon N. (2016). International Pediatric Otolaryngology Group (IPOG) consensus recommendations: Hearing loss in the pediatric patient. Int. J. Pediatr. Otorhinolaryngol..

[9] Shearer A.E., Hildebrand M.S., Smith R.J.H., Adam M.P., Ardinger H.H., Pagon R.A., Wallace S.E., Bean L.J.H., Mirzaa G., Amemiya A. (1993). Hereditary Hearing Loss and Deafness Overview.

[10] Smith R.H., Van Camp G. (2010). Deafness and Hereditary Hearing Loss Overview.

[11] Ahmadmehrabi S., Brant J., Epstein D.J., Ruckenstein M.J., Rader D.J. (2021). Genetics of Postlingual Sensorineural Hearing Loss. Laryngoscope.

[12] Richards S., Aziz N., Bale S., Bick D., Das S., Gastier-Foster J., Grody W.W., Hegde M., Lyon E., Spector E. (2015). Standards and guidelines for the interpretation of sequence variants: A joInt. consensus recommendation of the American College of Medical Genetics and Genomics and the Association for Molecular Pathology. Genet. Med..

[13] Krumm N., Sudmant P.H., Ko A., O’Roak B.J., Malig M., Coe B.P., Project N.E.S., Quinlan A.R., Nickerson D.A., Eichler E.E. (2012). Copy number variation detection and genotyping from exome sequence data. Genome Res..

[14] Fromer M., Purcell S.M. (2014). Using XHMM Software to Detect Copy Number Variation in Whole-Exome Sequencing Data. Curr. Protoc. Hum. Genet..

[15] Buels R., Yao E., Diesh C.M., Hayes R.D., Munoz-Torres M., Helt G., Goodstein D.M., Elsik C.G., Lewis S.E., Stein L. (2016). JBrowse: A dynamic web platform for genome visualization and analysis. Genome Biol..

[16] Chan D.K., Chang K.W. (2014). GJB2-associated hearing loss: Systematic review of worldwide prevalence, genotype, and auditory phenotype. Laryngoscope.

[17] Savige J., Colville D., Rheault M., Gear S., Lennon R., Lagas S., Finlay M., Flinter F. (2016). Alport Syndrome in Women and Girls. Clin. J. Am. Soc. Nephrol..

[18] Barker D.F., Hostikka S.L., Zhou J., Chow L.T., Oliphant A.R., Gerken S.C., Gregory M.C., Skolnick M.H., Atkin C.L., Tryggvason K. (1990). Identification of mutations in the COL4A5 collagen gene in Alport syndrome. Science.

[19] Jais J.P., Knebelmann B., Giatras I., De Marchi M., Rizzoni G., Renieri A., Weber M., Gross O., Netzer K.O., Flinter F. (2003). X-linked Alport syndrome: Natural history and genotype-phenotype correlations in girls and women belonging to 195 families: A “European Community Alport Syndrome Concerted Action” study. J. Am. Soc. Nephrol..

[20] Pennings R.J., Huygen P.L., Orten D.J., Wagenaar M., van Aarem A., Kremer H., Kimberling W.J., Cremers C.W., Deutman A.F. (2004). Evaluation of visual impairment in Usher syndrome 1b and Usher syndrome 2a. Acta Ophthalmol. Scand..

[21] Richard G., White T.W., Smith L.E., Bailey R.A., Compton J.G., Paul D.L., Bale S.J. (1998). Functional defects of Cx26 resulting from a heterozygous missense mutation in a family with dominant deaf-mutism and palmoplantar keratoderma. Hum. Genet..

[22] Uyguner O., Tukel T., Baykal C., Eris H., Emiroglu M., Hafiz G., Ghanbari A., Baserer N., Yuksel-Apak M., Wollnik B. (2002). The novel R75Q mutation in the GJB2 gene causes autosomal dominant hearing loss and palmoplantar keratoderma in a Turkish family. Clin. Genet..

[23] Ruf R.G., Xu P.X., Silvius D., Otto E.A., Beekmann F., Muerb U.T., Kumar S., Neuhaus T.J., Kemper M.J., Raymond R.M. (2004). SIX1 mutations cause branchio-oto-renal syndrome by disruption of EYA1-SIX1-DNA complexes. Proc. Natl. Acad. Sci. USA.

[24] Carminho-Rodrigues M.T., Klee P., Laurent S., Guipponi M., Abramowicz M., Cao-van H., Guinand N., Paoloni-Giacobino A. (2020). LARS2-Perrault syndrome: A new case report and literature review. BMC Med. Genet..

[25] Borck G., Ur Rehman A., Lee K., Pogoda H.M., Kakar N., von Ameln S., Grillet N., Hildebrand M.S., Ahmed Z.M., Nurnberg G. (2011). Loss-of-function mutations of ILDR1 cause autosomal-recessive hearing impairment DFNB42. Am. J. Hum. Genet..

[26] Kemerley A., Sloan C., Pfeifer W., Smith R., Drack A. (2017). A novel mutation in ACTG1 causing Baraitser-Winter syndrome with extremely variable expressivity in three generations. Ophthalmic Genet..

[27] Yuan Y., Gao X., Huang B., Lu J., Wang G., Lin X., Qu Y., Dai P. (2016). Phenotypic Heterogeneity in a DFNA20/26 family segregating a novel ACTG1 mutation. BMC Genet..

[28] Miyajima H., Moteki H., Day T., Nishio S.Y., Murata T., Ikezono T., Takeda H., Abe S., Iwasaki S., Takahashi M. (2020). Novel ACTG1 mutations in patients identified by massively parallel DNA sequencing cause progressive hearing loss. Sci. Rep..

[29] Van Esch H., Groenen P., Nesbit M.A., Schuffenhauer S., Lichtner P., Vanderlinden G., Harding B., Beetz R., Bilous R.W., Holdaway I. (2000). GATA3 haplo-insufficiency causes human HDR syndrome. Nature.

[30] Thirlwall A.S., Brown D.J., McMillan P.M., Barker S.E., Lesperance M.M. (2003). Phenotypic characterization of hereditary hearing impairment linked to DFNA25. Arch. Otolaryngol. Head Neck Surg..

[31] Riazuddin S.A., Parker D.S., McGlumphy E.J., Oh E.C., Iliff B.W., Schmedt T., Jurkunas U., Schleif R., Katsanis N., Gottsch J.D. (2012). Mutations in LOXHD1, a recessive-deafness locus, cause dominant late-onset Fuchs corneal dystrophy. Am. J. Hum. Genet..

[32] Shearer A.E., Smith R.J. (2015). Massively Parallel Sequencing for Genetic Diagnosis of Hearing Loss: The New Standard of Care. Otolaryngol. Head Neck Surg..

[33] Laurent S., Gehrig C., Nouspikel T., Amr S.S., Oza A., Murphy E., Vannier A., Bena F.S., Carminho-Rodrigues M.T., Blouin J.L. (2021). Molecular characterization of pathogenic OTOA gene conversions in hearing loss patients. Hum. Mutat..

[34] Lee K., Chiu I., Santos-Cortez R.L., Basit S., Khan S., Azeem Z., Andrade P.B., Kim S.S., Ahmad W., Leal S.M. (2013). Novel OTOA mutations cause autosomal recessive non-syndromic hearing impairment in Pakistani families. Clin. Genet..

[35] Valero R., Bannwarth S., Roman S., Paquis-Flucklinger V., Vialettes B. (2008). Autosomal dominant transmission of diabetes and congenital hearing impairment secondary to a missense mutation in the WFS1 gene. Diabet. Med..

[36] Vona B., Hofrichter M.A., Neuner C., Schroder J., Gehrig A., Hennermann J.B., Kraus F., Shehata-Dieler W., Klopocki E., Nanda I. (2015). DFNB16 is a frequent cause of congenital hearing impairment: Implementation of STRC mutation analysis in routine diagnostics. Clin. Genet..

[37] Kim H.J., Won H.H., Park K.J., Hong S.H., Ki C.S., Cho S.S., Venselaar H., Vriend G., Kim J.W. (2013). SNP linkage analysis and whole exome sequencing identify a novel POU4F3 mutation in autosomal dominant late-onset nonsyndromic hearing loss (DFNA15). PLoS ONE.

[38] Pesch U.E., Leo-Kottler B., Mayer S., Jurklies B., Kellner U., Apfelstedt-Sylla E., Zrenner E., Alexander C., Wissinger B. (2001). OPA1 mutations in patients with autosomal dominant optic atrophy and evidence for semi-dominant inheritance. Hum. Mol. Genet..

[39] Ham M., Han J., Osann K., Smith M., Kimonis V. (2019). Meta-analysis of genotype-phenotype analysis of OPA1 mutations in autosomal dominant optic atrophy. Mitochondrion.

[40] Yu-Wai-Man P., Griffiths P.G., Gorman G.S., Lourenco C.M., Wright A.F., Auer-Grumbach M., Toscano A., Musumeci O., Valentino M.L., Caporali L. (2010). Multi-system neurological disease is common in patients with OPA1 mutations. Brain.

[41] Boothe M., Morris R., Robin N. (2020). Stickler Syndrome: A Review of Clinical Manifestations and the Genetics Evaluation. J. Pers Med..

[42] Rad A., Schade-Mann T., Gamerdinger P., Yanus G.A., Schulte B., Muller M., Imyanitov E.N., Biskup S., Lowenheim H., Tropitzsch A. (2021). Aberrant COL11A1 splicing causes prelingual autosomal dominant nonsyndromic hearing loss in the DFNA37 locus. Hum. Mutat..

[43] Schepers D., Tortora G., Morisaki H., MacCarrick G., Lindsay M., Liang D., Mehta S.G., Hague J., Verhagen J., van de Laar I. (2018). A mutation update on the LDS-associated genes TGFB2/3 and SMAD2/3. Hum. Mutat..

[44] Riazuddin S., Khan S.N., Ahmed Z.M., Ghosh M., Caution K., Nazli S., Kabra M., Zafar A.U., Chen K., Naz S. (2006). Mutations in TRIOBP, which encodes a putative cytoskeletal-organizing protein, are associated with nonsyndromic recessive deafness. Am. J. Hum. Genet..

[45] Wattenhofer M., Di Iorio M.V., Rabionet R., Dougherty L., Pampanos A., Schwede T., Montserrat-Sentis B., Arbones M.L., Iliades T., Pasquadibisceglie A. (2002). Mutations in the TMPRSS3 gene are a rare cause of childhood nonsyndromic deafness in Caucasian patients. J. Mol. Med..

[46] Rosado C., Bueno E., Fraile P., Garcia-Cosmes P., Gonzalez-Sarmiento R. (2015). A new mutation in the COL4A3 gene responsible for autosomal dominant Alport syndrome, which only generates hearing loss in some carriers. Eur. J. Med. Genet..

[47] Nayak G., Varga L., Trincot C., Shahzad M., Friedman P.L., Klimes I., Greinwald J.H., Riazuddin S.A., Masindova I., Profant M. (2015). Molecular genetics of MARVELD2 and clinical phenotype in Pakistani and Slovak families segregating DFNB49 hearing loss. Hum. Genet..

[48] Zhang J., Guan J., Wang H., Yin L., Wang D., Zhao L., Zhou H., Wang Q. (2019). Genotype-phenotype correlation analysis of MYO15A variants in autosomal recessive non-syndromic hearing loss. BMC Med. Genet..

[49] Kresak J.L., Walsh M. (2016). Neurofibromatosis: A Review of NF1, NF2, and Schwannomatosis. J. Pediatr. Genet..

[50] Kemperman M.H., Bom S.J., Lemaire F.X., Verhagen W.I., Huygen P.L., Cremers C.W. (2002). DFNA9/COCH and its phenotype. Adv. Otorhinolaryngol..

[51] Chakchouk I., Grati M., Bademci G., Bensaid M., Ma Q., Chakroun A., Foster J., Yan D., Duman D., Diaz-Horta O. (2015). Novel mutations confirm that COL11A2 is responsible for autosomal recessive non-syndromic hearing loss DFNB53. Mol. Genet. Genom..

[52] Balestrini S., Milh M., Castiglioni C., Luthy K., Finelli M.J., Verstreken P., Cardon A., Strazisar B.G., Holder J.L., Lesca G. (2016). TBC1D24 genotype-phenotype correlation: Epilepsies and other neurologic features. Neurology.

[53] Azaiez H., Booth K.T., Bu F., Huygen P., Shibata S.B., Shearer A.E., Kolbe D., Meyer N., Black-Ziegelbein E.A., Smith R.J. (2014). TBC1D24 mutation causes autosomal-dominant nonsyndromic hearing loss. Hum. Mutat..

[54] Astuto L.M., Bork J.M., Weston M.D., Askew J.W., Fields R.R., Orten D.J., Ohliger S.J., Riazuddin S., Morell R.J., Khan S. (2002). CDH23 mutation and phenotype heterogeneity: A profile of 107 diverse families with Usher syndrome and nonsyndromic deafness. Am. J. Hum. Genet..

[55] Jouret G., Poirsier C., Spodenkiewicz M., Jaquin C., Gouy E., Arndt C., Labrousse M., Gaillard D., Doco-Fenzy M., Lebre A.S. (2019). Genetics of Usher Syndrome: New Insights From a Meta-analysis. Otol. Neurotol..

[56] Ahmed Z.M., Riazuddin S., Aye S., Ali R.A., Venselaar H., Anwar S., Belyantseva P.P., Qasim M., Riazuddin S., Friedman T.B. (2008). Gene structure and mutant alleles of PCDH15: Nonsyndromic deafness DFNB23 and type 1 Usher syndrome. Hum. Genet..

[57] Toualbi L., Toms M., Moosajee M. (2020). USH2A-retinopathy: From genetics to therapeutics. Exp. Eye Res..

[58] Thoenes M., Zimmermann U., Ebermann I., Ptok M., Lewis M.A., Thiele H., Morlot S., Hess M.M., Gal A., Eisenberger T. (2015). OSBPL2 encodes a protein of inner and outer hair cell stereocilia and is mutated in autosomal dominant hearing loss (DFNA67). Orphanet J. Rare Dis..

[59] Sommen M., Wuyts W., Van Camp G. (2017). Molecular diagnostics for hereditary hearing loss in children. Expert Rev. Mol. Diagn..

[60] Downie L., Amor D.J., Halliday J., Lewis S., Martyn M., Goranitis I. (2021). Exome Sequencing for Isolated Congenital Hearing Loss: A Cost-Effectiveness Analysis. Laryngoscope.

[61] Zhao M., Wang Q., Wang Q., Jia P., Zhao Z. (2013). Computational tools for copy number variation (CNV) detection using next-generation sequencing data: Features and perspectives. BMC Bioinform..

[62] Park J.H., Kim N.K., Kim A.R., Rhee J., Oh S.H., Koo J.W., Nam J.Y., Park W.Y., Choi B.Y. (2014). Exploration of molecular genetic etiology for Korean cochlear implantees with severe to profound hearing loss and its implication. Orphanet J. Rare Dis..

[63] Chen S., Dong C., Wang Q., Zhong Z., Qi Y., Ke X., Liu Y. (2016). Targeted Next-Generation Sequencing Successfully Detects Causative Genes in Chinese Patients with Hereditary Hearing Loss. Genet. Test. Mol. Biomark..

[64] Iwasa Y.I., Nishio S.Y., Usami S.I. (2016). Comprehensive Genetic Analysis of Japanese Autosomal Dominant Sensorineural Hearing Loss Patients. PLoS ONE.

[65] Christensen K., Frederiksen H., Hoffman H.J. (2001). Genetic and environmental influences on self-reported reduced hearing in the old and oldest old. J. Am. Geriatr. Soc..

[66] Lewis M.A., Nolan L.S., Cadge B.A., Matthews L.J., Schulte B.A., Dubno J.R., Steel K.P., Dawson S.J. (2018). Whole exome sequencing in adult-onset hearing loss reveals a high load of predicted pathogenic variants in known deafness-associated genes and identifies new candidate genes. BMC Med. Genom..

[67] Zazo Seco C., Wesdorp M., Feenstra I., Pfundt R., Hehir-Kwa J.Y., Lelieveld S.H., Castelein S., Gilissen C., de Wijs I.J., Admiraal R.J. (2017). The diagnostic yield of whole-exome sequencing targeting a gene panel for hearing impairment in The Netherlands. Eur. J. Hum. Genet..

[68] Morin M., Bryan K.E., Mayo-Merino F., Goodyear R., Mencia A., Modamio-Hoybjor S., del Castillo I., Cabalka J.M., Richardson G., Moreno F. (2009). In vivo and in vitro effects of two novel γ-actin (ACTG1) mutations that cause DFNA20/26 hearing impairment. Hum. Mol. Genet..

[69] Battelino S., Klancar G., Kovac J., Battelino T., Trebusak Podkrajsek K. (2016). TMPRSS3 mutations in autosomal recessive nonsyndromic hearing loss. Eur. Arch. Otorhinolaryngol..

[70] Sloan-Heggen C.M., Bierer A.O., Shearer A.E., Kolbe D.L., Nishimura C.J., Frees K.L., Ephraim S.S., Shibata S.B., Booth K.T., Campbell C.A. (2016). Comprehensive genetic testing in the clinical evaluation of 1119 patients with hearing loss. Hum. Genet..

[71] Rudman J.R., Kabahuma R.I., Bressler S.E., Feng Y., Blanton S.H., Yan D., Liu X.Z. (2017). The genetic basis of deafness in populations of African descent. J. Genet. Genom..

[72] Wu C.C., Tsai C.Y., Lin Y.H., Chen P.Y., Lin P.H., Cheng Y.F., Wu C.M., Lin Y.H., Lee C.Y., Erdenechuluun J. (2019). Genetic Epidemiology and Clinical Features of Hereditary Hearing Impairment in the Taiwanese Population. Genes.

[73] Smith E.D., Blanco K., Sajan S.A., Hunter J.M., Shinde D.N., Wayburn B., Rossi M., Huang J., Stevens C.A., Muss C. (2019). A retrospective review of multiple findings in diagnostic exome sequencing: Half are distinct and half are overlapping diagnoses. Genet. Med..

