# Supplementary material for: Benefits of Exome Sequencing in Children with Suspected Isolated Hearing Loss
Source: Genes (Basel). 2021 Aug 20;12(8):1277. doi: 10.3390/genes12081277 (PMC8391342; doi:10.3390/genes12081277)
Supplement: Supplementary file 1 [file genes-12-01277-s001.zip › Table S1 In house hearing loss gene panel-RV.pdf]

Table S1. In house hearing loss gene panel (189 genes). Genes are listed following their HUGO Gene Nomenclature Commitee symbol.

ABCC1	COL11A1	FRAS1	MAN2B1	PAX3	SOX10
ABHD12	COL11A2	FOXI1	MANBA	PCDH15	SOX2
ABHD5	COL2A1	FREM2	MARVELD2	PDZD7	STRC
ACTG1	COL4A3	FXN	MASP1	PEX1	SYNE4
ADCY1	COL4A4	GATA3	MET	PJVK	TBC1D24
ADGRV1	COL4A5	GIPC3	MITF	PLCB4	TCOF1
AIFM1	COL4A6	GJB2	MSRB3	PNPT1	TECTA
ALMS1	COL9A1	GJB3	MT-RNR1	POLR1A	TFAP2A
ANKH	COL9A2	GJB6	MT-TE	POLR1C	TIMM8A
ATP6V1B1	COL9A3	GNAI3	MT-TK	POLR1D	TJP2
BCS1L	CRYM	GPSM2	MT-TL1	POU3F4	TMC1
BDP1	DCDC2	GRHL2	MT-TS1	POU4F3	TMEM132E
BSND	DHODH	GRIP1	MYH14	PRPS1	TMIE
BTD	DIABLO	GRXCR1	MYH9	PTPRQ	TMPRSS3
CABP2	DIAPH1	GSC	MYO15A	RDX	TNC
CACNA1D	DIAPH3	GRXCR2	MYO1A	RIPOR2	TPRN
CCDC50	DNMT1	GSDME	MYO3A	RPS6KA3	TRIOBP
CDC6	DSPP	HARS2	MYO6	SALL1	TSPEAR
CD151	EDN3	HGF	MYO7A	SALL4	TYR
CDH23	EDNRA	HMX1	NARS2	SEMA3E	USH1C
CDT1	EDNRB	HOMER2	NDP	SERAC1	USH1G
CEACAM16	EFTUD2	HOXA2	NLRP12	SERPINB6	USH2A
CEP78	EIF4A3	HSD17B4	NLRP3	SIX1	WFS1
CHD7	ELMOD3	ILDR1	OPA1	SIX5	WHRN
CHSY1	EPS8	KARS	ORC1	SLC17A8	
CIB2	ESPN	KCNE1	ORC4	SLC19A2	
CISD2	ESRRB	KCNJ10	ORC6	SLC26A4	
CLDN14	EYA1	KCNQ1	OSBPL2	SLC26A5	
CLIC5	EYA4	KCNQ4	OTOA	SLC29A3	
CLPP	FGF10	LARS2	OTOF	SLC4A11	
CLRN1	FGF3	LHFPL5	OTOG	SLITRK6	
ABCC1	FGFR2	LOXHD1	OTOGL	SMPX	
COCH	FGFR3	LRTOMT	P2RX2	SNAI2	
