# Supplementary material for: Benefits of Exome Sequencing in Children with Suspected Isolated Hearing Loss
Source: Genes (Basel). 2021 Aug 20;12(8):1277. doi: 10.3390/genes12081277 (PMC8391342; doi:10.3390/genes12081277)
Supplement: Supplementary file 1 [file genes-12-01277-s001.zip › Table S2 phenotype children_RV.pdf]

Table S2. **Phenotypes in children**: brief description of auditory phenotypes in our child cohort.

ADHD= attention deficit hyperactivity disorder, ASD= autism spectrum disorder, CP= cleft palate, CT=computed tomography, F= female, IUGR= in utero growth retardation, M=male, M=magnetic resonance imaging, NR= not reported, SN= sensorineural, VSD = ventricular septal defect.

Patient	Sex	Age at diagnosis	Type	Laterality	Severity	Progressive	Family history	Consanguinity	Malformations at the time of CT/MRI	Other
1	F	Prelingual	SN	Bilateral	Moderate	No	No	No	Yes	No
2	F	Congenital	SN	Bilateral	Profound	No	No	No	No	NR
3	M	Congenital	SN	Bilateral	Moderate	No	No	No	No	NR
4	M	Congenital	Mixed	Bilateral	Moderate	No	No	No	Yes	Preauricular pit/ cholesteatoma
5	F	Postlingual	SN	Bilateral	Profound	No	No	No	No	NR
6	M	Congenital	SN	Bilateral	Profound	No	No	Yes	Not performed	NR
7	M	Postlingual	Mixed	Unilateral	Mild	No	No	No	Yes	NR
8	M	Congenital	SN	Bilateral	Moderate	No	No	No	No	NR
9	M	Congenital	SN	Bilateral	Moderate	No	No	No	Not performed	Renal cysts/relational issues/ASD -like features
10	M	Congenital	SN	Bilateral	Moderate	No	Yes	No	Not performed	NR
11	F	Congenital	SN	Bilateral	Moderate	No	No	No	Yes	NR
12	M	Postlingual	SN	Bilateral	Mild	No	No	No	No	NR
13	M	Postlingual	SN	Bilateral	Moderate	No	No	No	No	NR
14	F	Prelingual	SN	Bilateral	Moderate	No	No	No	Not performed	NR
15	M	Congenital	SN	Bilateral	Moderate	No	No	No	Not performed	NR
16	F	Postlingual	SN	Bilateral	Moderate	No	No	No	No	NR
17	F	Congenital	SN	Bilateral	Moderate	No	No	Yes	No	NR
18	F	Congenital	SN	Bilateral	Moderate	Yes	No	No	No	Optic atrophy/ IUGR/atrial septal defect

19	M	Congenital	SN	Bilateral	Moderate	No	No	No	Not performed	Unilateral renal dysplasia
20	M	Congenital	SN	Bilateral	Severe	No	No	No	Yes	Keratoderma palmoplantar
21	F	Postlingual	SN	Bilateral	Moderate	No	Yes	No	Not performed	No
22	F	Congenital	SN	Bilateral	Moderate	No	Yes	No	Not performed	NR
23	M	Prelingual	SN	Bilateral	Mild	No	Yes	No	Not performed	Excessive timidity/limited interactions
24	F	Congenital	SN	Bilateral	Moderate	No	no	No	Not performed	CP /myopia - patella luxation/ arachnodactyly
25	F	Congenital	SN	Bilateral	Severe	No	No	Yes	No	NR
26	M	Congenital	SN	Bilateral	Moderate	No	Yes	No	Not performed	NR
27	F	Congenital	SN	Bilateral	Severe	Yes	No	No	Not performed	NR
28	M	Postlingual	SN	Bilateral	Mild	No	Yes	No	No	NR
29	M	Prelingual	SN	Bilateral	Severe	Yes	Yes	No	No	NR
30	M	Postlingual	SN	Bilateral	Moderate	Yes	Yes	No	Not performed	NR
31	M	Prelingual	SN	Bilateral	Severe	No	No	No	Not performed	Cholestasis /ADHD
32	M	Congenital	SN	Bilateral	Profound	No	Yes	No	Yes	Atrial septal defect/ hypothyroidism
33	M	Prelingual	SN	Bilateral	Severe	No	Yes	No	No	NR
34	M	Congenital	SN	Bilateral	Mild	No	Yes	No	Not performed	NR
35	M	Prelingual	SN	Bilateral	Moderate	Yes	No	No	No	NR
36	M	Congenital	SN	Unilateral	Profound	No	No	No	No	NR
37	F	Prelingual	Mixed	Bilateral	Moderate	No	No	No	Yes	No
38	F	Congenital	SN	Bilateral	Mild	No	Yes	No	No	NR
39	M	Congenital	Mixed	Bilateral	Moderate	No	No	No	Yes	Vertebral malformations
40	F	Postlingual	SN	Bilateral	Moderate	No	Yes	No	No	NR
41	M	Congenital	SN	Unilateral	Profound	No	No	No	No	Heterochromia iridis

42	F	Congenital	SN	Bilateral	Severe	No	No	No	No	NR
43	M	Congenital	Mixed	Bilateral	Moderate	No	No	No	Yes	NR
44	M	Congenital	SN	Bilateral	Profound	No	No	No	Yes	Vestibular areflexia
45	M	Postlingual	SN	Unilateral	Moderate	No	No	No	Not performed	Unilateral renal dysplasia
46	M	Postlingual	SN	Bilateral	Severe	No	Yes	No	No	NR
47	M	Congenital	SN	Bilateral	Profound	No	No	No	Not performed	Asperger syndrome
48	F	Postlingual	Mixed	Unilateral	Moderate	No	Yes	No	Yes	NR
49	F	Postlingual	SN	Unilateral	Moderate	No	No	No	No	NR
50	M	Postlingual	SN	Bilateral	Moderate	Yes	Yes	No	Yes	NR
51	F	Congenital	SN Auditory neuropathy	Bilateral	Moderate	No	No	No	No	NR
52	M	Congenital	SN	Bilateral	Moderate	No	no	No	No	NR
53	M	Congenital	SN	Bilateral	Mild	No	Yes	No	Yes	Dysplasic helix /VSD and atrial septal defect - micropenis / hypospadias/ right testicular hypoplasia /left kidney duplication/ teeth malposition
54	F	Prelingual	SN	Bilateral	Severe	No	No	No	Not performed	Walking delay
55	M	Prelingual	SN	Bilateral	Moderate	No	No	No	No	IUGR /global developmental delay
56	M	Congenital	SN (right) mixed (left)	Bilateral	Moderate	No	No	No	Not performed	Global developmental delay-/VSD/inguinal hernia /CP /facial asymmetry/ bilateral atreasia of the auditory canals/ lop ears/ right auricular appendix

57	F	Congenital	Transmission	Unilateral	Mild	No	No	No	Yes	Overgrowth/motor developmental delay/hypotonia /global hyperlaxity/macrocephaly/ downslanting palpebral fissures/ strabism / dysplasic ears/bifid uvula / VSD /flat feet
58	F	Prelingual	Transmission	Bilateral	Moderate	No	Yes	Yes	Yes	Narrow external auditory canal/ CP /right ear cholesteatoma
59	F	Congenital	SN	Bilateral	Moderate	No	No	No	No	Speech delay
60	F	Congenital	SN	Unilateral	Severe	No	Yes	No	No	No
61	F	Congenital	SN	Bilateral	Moderate	No	Yes	No	Not performed	No
