# Supplementary material for: Benefits of Exome Sequencing in Children with Suspected Isolated Hearing Loss
Source: Genes (Basel). 2021 Aug 20;12(8):1277. doi: 10.3390/genes12081277 (PMC8391342; doi:10.3390/genes12081277)
Supplement: Supplementary file 1 [file genes-12-01277-s001.zip › Table S3 phenotype adults_RV.pdf]

Table S3. **Phenotypes in adults**: brief description of auditory and associated phenotypes in our adult cohort.

AMD= age-related macular degeneration, CT=computed tomography, F= female, HL=hearing loss, M=male, MRI=magnetic resonance imaging, SN= sensorineural.

Patient	Sex	Age at diagnosis	Age and type of onset	Type	Laterality	Severity	Progressive	Family history	Consanguinity	Malformations at the time of CT/MRI	Other
62	M	Postlingual	29 years - progressive and asymmetric	SN	Bilateral (asymmetric)	Moderate	Yes	Yes	No	Yes	Multiple mengiomes
63	F	Postlingual	30 years - progressive	SN	Bilateral	Severe	Yes	Yes	No	Yes	Vertigo / vestibular areflexia
64	F	Postlingual	8 years – hearing aids at 18 years - progressive	SN	Bilateral	Moderate	Yes	Yes	Yes	No	No
65	F	Postlingual	Started brutally at 52 years with vertigo - first profound right ear HL, followed by left ear HL	Perception	Bilateral (asymmetric)	Severe	Yes	No	Yes	Yes	Vertigo /right vestibular areflexia
66	M	Postlingual	65 years - progressive	SN	Bilateral (asymmetric)	Moderate	Yes	No	No	Yes	AMD
67	M	Postlingual	Childhood - progressive	SN	Bilateral	Severe	Yes	Yes	No	Not performed	No

68	F	Postlingual	45 years – sudden HL after upper airway infection - left > right	SN	Bilateral	Profound	Yes	No	No	No	Tinnitus
69	F	Postlingual	46 years - progressive first, then sudden worsening HL	SN	Bilateral	Severe	Yes	Unknown	Unknown	No	Vertigo/ white matter anomaly
70	F	Postlingual	Childhood - progressive - 2006 marked inconvenience	SN	Bilateral	Moderate	Yes	Yes	No	Not performed	No
