# Supplementary material for: Benefits of Exome Sequencing in Children with Suspected Isolated Hearing Loss
Source: Genes (Basel). 2021 Aug 20;12(8):1277. doi: 10.3390/genes12081277 (PMC8391342; doi:10.3390/genes12081277)
Supplement: Supplementary file 1 [file genes-12-01277-s001.zip › Table S4 Molecular results RV final.pdf]

Table S4. Summary of positive molecular results (children and adults (patient 62 and 63)). AD = autosomal dominant, AR= autosomal recessive, HL= hearing loss, LDS= Loeys-Dietz Syndrome, L.P. = Likely Pathogenic, MIM= Mendelian Inheritance in Man, N.A.= not applicable, p= protein, P.=Pathogenic, Pt.=Patient, rs= Reference SNP, XL = X-linked.

Gene ID	Molecular Results	Rs Number	ACMG classification	Transmission type	Phenotype (Phenotype MIM number)	Syndromic	Inheritance	Pt.
COL4A5	heterozygous c.1525G>C, p.(Gly509Arg)	N.A.	L.P. (class IV)	XL	Alport syndrome, 1 X-linked (MIM: 301050)	Yes potentially	*De novo*	*1*
USH1G	homozygous c.1373A>T, p.(Asp458Val)	rs397517925	L.P. (class IV)	AR	Usher syndrome, 1G (MIM: 606943)	Yes	Inherited in trans	*2*
GJB2	compound heterozygous for c.35delG, p.(Gly12Valfs*2) ; heterozygous c.101T>C, p.(Met34Thr)	rs80338939, rs35887622	P. (class V)/P. (class V)	AR	Deafness, autosomal recessive 1A (MIM: 220290)	No	Inherited in trans	*3*
GJB2	compound heterozygous c.35del, p.(Gly12Valfs*2) ; c.139G>T, p.(Glu47*)	rs80338939, rs104894398	P. (class V)/P. (class V)	AR	Deafness, autosomal recessive 1A (MIM: 220290)	No	Inherited in trans	*8*
GJB2	heterozygous c. 223C>T, p.(Arg75Trp)	rs104894402	P. (class V)	AD	Keratoderma, palmoplantar, with deafness (MIM: 148350)	Yes	*De novo*	*20*
SIX1	heterozygous c.386A>C, p.(Tyr129Ser)	rs104894478	P. (class V)	AD	Branchiootic syndrome, 3 (MIM: 608389) ; Deafness, autosomal dominant 23 (MIM:605192)	Yes	*De novo*	*4*

LARS2	compound heterozygous c.457A>C, p.(Asn153His); c.1565C>A, p.(Thr522Asn)	rs786205560, rs199589947	L.P. (class IV) / P. (class V)	AR	Perrault syndrome, 4 (MIM: 615300)	Yes ovarian failure diagnosed after molecular diagnosis	Inherited in trans	*5*
ILDR1	homozygous c.942C>A, p.(Cys314*)	rs752714222	P. (class V)	AR	Deafness, autosomal recessive 42 (MIM: 609646)	No	Inherited in trans	*6*
ACTG1	heterozygous c.440G>A, p.(Arg147His)	N.A.	L.P. (class IV)	AD	Deafness, autosomal dominant 20/26 (MIM: 604717)	No	*De novo*	*7*
ACTG1	heterozygous c.826G>A, p.(Glu276Lys)	N.A.	L.P. (class IV)	AD	Deafness, autosomal dominant 20/26 (MIM: 604717)	No	*De novo*	*16*
ACTG1	heterozygous c.830C>T p.(Thr277Ile)	N.A.	P. (class V)	AD	Deafness, autosomal dominant progressive 20/26 (MIM: 604717), Baraitser-Winter syndrome (MIM: 614853)	Yes potentially	*De novo*	*30*
GATA3	heterozygous c.778+1G>A, p.(?)	N.A.	P. (class V)	AD	Hypoparathyroidism, sensorineural deafness, and renal dysplasia (MIM: 146255)	Yes	*De novo*	*9*
GATA3	heterozygous c.431delG, p.(Gly144Alafs*51)	rs1588377948	P. (class V)	AD	Hypoparathyroidism, sensorineural deafness, and renal dysplasia (MIM: 146255)	Yes	*De novo*	*19*
SLC17A8	heterozygous c.634C>A, p.(Pro212Thr)	N.A.	L.P. (class IV)	AD	Deafness, autosomal dominant 25 (MIM: 605583)	No	Inherited from mother without HL	*10*
LOXHD1	homozygous c.3061+1G>A, p.(?)	rs537227442	P. (class V)	AR	Deafness, autosomal recessive 77 (MIM: 613079)	No	Inherited one variant from mother/father N.A.	*11*

OTOA	Compound heterozygous for a gene conversion between *OTOA* gene and *OTOAP1* pseudogene; deletion of *OTOA*	N.A.	P. (class V)	AR	Deafness, autosomal recessive 22 (MIM: 607039)	No	Inherited in trans	*17*
WFS1	heterozygous c.2051C>T, p.(Ala684Val)	rs387906930	P. (class V)	AD	Wolfram-like syndrome, autosomal dominant (MIM: 614296)	Yes optic atrophy discovered after molecular diagnosis	*De novo*	*18*
STRC	compound heterozygous *CKMT1B, STRC, CATSPER2* deletion ; c.4917_4918delACinsCT, p.(Leu1640Phe) in *STRC*	N.A./ rs727503441	P.(class V)/ V.U.S (class III)	AR	Deafness, autosomal recessive 16 (MIM: 603720)	No	Inherited in trans	*12*
STRC	compound heterozygous *CKMT1B, STRC* deletion; *CKMT1B, STRC, CATSPER2* deletion	N.A.	P. (class V)/P. (class V)	AR	Deafness, autosomal recessive 16 (MIM: 603720)	No	Inherited in trans	*13*

STRC	compound heterozygous c.4425G>C, p.(Trp1475Cys) in *STRC* and *CKMT1B, STRC, CATSPER2* deletion	rs727503443/ N.A.	L.P. (class IV)/ P. (class V)	AR	Deafness, autosomal recessive 16 (MIM: 603720)	No	Inherited in trans	*14*
STRC ; CKMT1B ; CATSPER2	homozygous deletion of *CKMT1B, STRC, CATSPER2*	N.A.	P. (class V)/P. (class V)	AR	Deafness and male infertility (MIM: 611102)	Yes potentially	Inherited in trans	*15*
STRC	compound heterozygous c.4837G>T, p.(Glu1613*)in *STRC* and *CKMT1B, STRC,* (and maybe *CATSPER2*) deletion	rs769443188/ N.A.	P. (class V)/P. (class V)	AR	Deafness, autosomal recessive 16 (MIM: 603720)	No	Inherited in trans	*22*
POU4F3; OPA1	heterozygous c.502del, p.(Ala168Profs*36) in *POU4F3;* heterozygous c.1118C>G, p.(Ser373Cys) in *OPA1*	rs766631025/ N.A.	P. (class V)/ L.P. (class IV)	AD	Deafness, autosomal dominant 15 (MIM: 602459); Optic atrophy plus syndrome (MIM: 125250).	Yes potentially	POU4F3 Inherited from father with HL/OPA1 *de novo*	*21*
COL11A1	heterozygous deletion	N.A.	P. (class V)	AD	Deafness, autosmal dominant 37 (MIM 618533) Stickler Syndrome II (MIM: 604841) / Marshall syndrome (MIM: 154780)	Yes potentially	Inherited from mother with HL	*23*

COL11A1	heterozygous deletion splicing site *COL11A1*, (c.4519-2Adel,p.(?))	N.A.	L.P. (class IV)	AD	Deafness, autosmal dominant 37 (MIM 618533) Stickler Syndrome II (MIM: 604841) / Marshall syndrome (MIM: 154780)	Yes potentially	Inherited from mother without HL	*26*
COL11A1; SMAD3	heterozygous c.4547G>T, p.(Gly1516Val) in *COL11A1;* heterozygous c.3G>A (p.Met1?) in *SMAD3*	rs1553193910 /N.A.	P. (class V)/ L.P. (class IV)	AD	Deafness, autosmal dominant 37 (MIM 618533) Stickler Syndrome II (MIM: 604841) / Marshall syndrome (MIM: 154780); Loeys-Dietz syndrome (MIM:613795)	Yes for Stickler syndrome/ potentially for LDS	SMAD3 inherited from affected mother/COLL1A1 *de novo*	*24*
TRIOBP	homozygous c.3214dup, p.(Arg1072Profs*12)	N.A.	P. (class V)	AR	Deafness, autosomal recessive 28 (MIM: 609823)	No	N.A.	*25*
TMPRSS3	compound heterozygous c.400A>T (p.LYS134*); c.646C>T (p.Arg216Cys)	N.A /N.A.	P. (class V)/P. (class V)	AR	Deafness, autosomal recessive 8 (MIM: 601072)	No	Inherited in trans	*27*
TMPRSS3	compound heterozygous c.916G>A p.(Ala306Thr); c.749delT p.(Leu250Argfs*25)	rs181949335/ N.A	P. (class V)/P. (class V)	AR	Deafness, autosomal recessive 8 (MIM: 601072)	No	Inherited in trans	*29*
COL4A3	heterozygous c.4826G>A, p.(Arg1609Gln)	rs1380878336	L.P. (class IV)	AD	Alport syndrome, 3 autosomal dominant (MIM: 104200)	Yes potentially	Inherited from father with HL and no sign of	*28*

							altered kidney function	
MarvelD2	homozygous c.1331+2T>C p.(?)	rs762352115	P. (class V)	AR	Deafness, autosmal recessive 49 (MIM: 610153)	No	Inherited in trans	*31*
MYO15A	homozygous c.6046+1G>A, p.(?)	rs201978571	P. (class V)	AR	Deafness, autosomal recessive 3 (MIM: 600316)	No	Inherited in trans	*32*
NF2	heterozygous c.1579 G>T, p.(Glu527*)	rs74315505	P. (class V)	AD	Neurofirbomatose type 2 (MIM: 101000)	Yes	N.A.	*62*
COCH	heterozygous c.341T>C , p.(Leu114Pro)	N.A.	P. (class V)	AD	Deafness, autsomal dominant 9 (MIM: 601369)	No	N.A.	*63*
