# Supplementary material for: Benefits of Exome Sequencing in Children with Suspected Isolated Hearing Loss
Source: Genes (Basel). 2021 Aug 20;12(8):1277. doi: 10.3390/genes12081277 (PMC8391342; doi:10.3390/genes12081277)
Supplement: Supplementary file 1 [file genes-12-01277-s001.zip › Table S5 Molecular results VUS-RV.pdf]

Table S5. Summary of molecular results classified as VUS (children and adults). Rs number are provided when available. MIM= *Mendelian Inheritance in Man*, rs= Reference SNP

Patient	Variant of unknown significance	Phenotype (Phenotype MIM number)	Heritance status
1	compound heterozygous c.5000G>A, p.(Arg1667His), rs146555195; c.4550A>G, p.(Asp1517Gly) in *COL11A2*	Deafness, autosomal recessive 53 (MIM: 609706)	Inherited in trans
33	compound heterozygous c.641G>A, p.(Arg214His), rs200324356; c.643T>G, p.(Trp215Gly), rs761347854 in *TBC1D24*	Deafness, autosomal recessive 86 (MIM: 614617)	Inherited in trans - affected brother carries both variants
42	heterozygous c.6841G>A, p.(Val228Ile) in *CDH23*	Deafness, autosomal recessive (MIM 601386)/ (Pituitary adenoma 5, multiple types) (MIM 617540)/ Autosomal dominant Usher syndrome 1D (MIM 601067), Autosmal recessive, Digenic recessive Usher syndrome Type 1D/F (601067)	Parents unavailable
46	heterozygous c.4885delA, p.S1629fs & c.964T>A, p.Ser322Thr in *PCDH15*;(cis) c.13133C>T, p.Pro437Leu & c.6800C>T, p.Pro2267 Leu, rs397518027 in *USH2A* (cis)	*PCDH15*: Deafness, autosomal recessive 23, autosomal recessive Usher syndrome 1D/F digenic (MIM 601607), Autosomal recessive, Digenic recessive/Usher syndrome 1F (MIM 602083)<br>*USH2A*: Autosomal recessive Usher syndrome, type 2A (MIM 276901), Retinitis pigmentosa 38 (MIM 613809)	All variants inherited from mother without HL
51	heterozygous c.852_854delTATinsATG, p.(Phe284_Met285delinsLeuTrp) in *OSBPL2*	Deafness, autosomal dominant (MIM: 616340)	Inherited from mother without HL
64	heterozygous c.418C>G, p.(Leu140Val) in *TBC1D24*	Deafness, autosomal dominant 65 (MIM: 616044)	Parents unavailable
