# Supplementary material for: Benefits of Exome Sequencing in Children with Suspected Isolated Hearing Loss
Source: Genes (Basel). 2021 Aug 20;12(8):1277. doi: 10.3390/genes12081277 (PMC8391342; doi:10.3390/genes12081277)
Supplement: Supplementary file 1 [file genes-12-01277-s001.zip › Table S6 GJB2 direct sequencing - RV.pdf]

Table S6. **GJB2 direct sequencing** – Brief description of patient's phenotype and variants in GJB2 detected by direct sequencing. All patients displayed congenital, SN, bilateral HL. None of them were born from a consanguinous union. F= female, M=male, NR= not reported, rs= Reference SNP.

Patient	Sex	Severity	Family history	Malformations at the CT/IRM	Additional signs	Variant description	Rs number
71	M	Severe	No	Yes	NR	homozygous c.35delG, p.(Gly12Valfs*2) in *GJB2*	rs80338939
72	M	Severe	No	Not performed	NR	homozygous c.35delG, p.(Gly12Valfs*2) in *GJB2*	rs80338939
73	F	Moderate	No	Not performed	NR	compound heterozygous for c.59T>C, p.(Ile20Thr), heterozygous for c.109G>A, p.(Val37Ile) in *GJB2*	rs1057517519; rs146378222
74	M	Severe	No	Yes	No	homozygous c.269T>C, p.Leu90Pro in *GJB2*	rs80338945
75	F	Profound	Yes	Not performed	No	homozygous c.35delG, p.(Gly12Valfs*2) in *GJB2*	rs80338939
